# The influence of cell membrane and SNAP25 linker loop on the dynamics and unzipping of SNARE complex

**DOI:** 10.1371/journal.pone.0176235

**Published:** 2017-04-20

**Authors:** Yi Shi, Yong Zhang, Jizhong Lou

**Affiliations:** 1 School of Life Sciences, University of Science and Technology of China, Hefei, Anhui, China; 2 Key Laboratory of RNA Biology, CAS Center for Excellence in Biomacromolecules, Institute of Biophysics, Chinese Academy of Sciences, Beijing, China; University of Illinois at Urbana-Champaign, UNITED STATES

## Abstract

The soluble N-ethylmaleimide-sensitive factor attachment protein receptor (SNARE) complex is composed of three neuronal proteins VAMP2, Syntaxin and SNAP25, which plays a core role during the process of membrane fusion. The zipping assembly of the SNARE complex releases energies and drives the vesicle and cell membrane into close proximity. In this study, we use all-atom molecular dynamics simulations to probe the dynamics of SNARE and its unzipping process in the context of membrane at the atomistic details. Our results indicated that the NTD of SNARE core domain is relatively more stable than CTD, which is in agreement with previous experiments. More importantly, possible interactions between the linker loop (LL) region of SNAP25 and VAMP2 are observed, suggests that the LL region may facilitate VAMP2 binding and SNARE initiation. The forced unzipping of SNARE in the presence of membrane and LL of SNAP25 reveals the possible pathway for energy generation of SNARE zipping, provides information to understand how force may regulate the cooperativity between the membrane and the SNARE complex.

## Introduction

**Fig 1:**
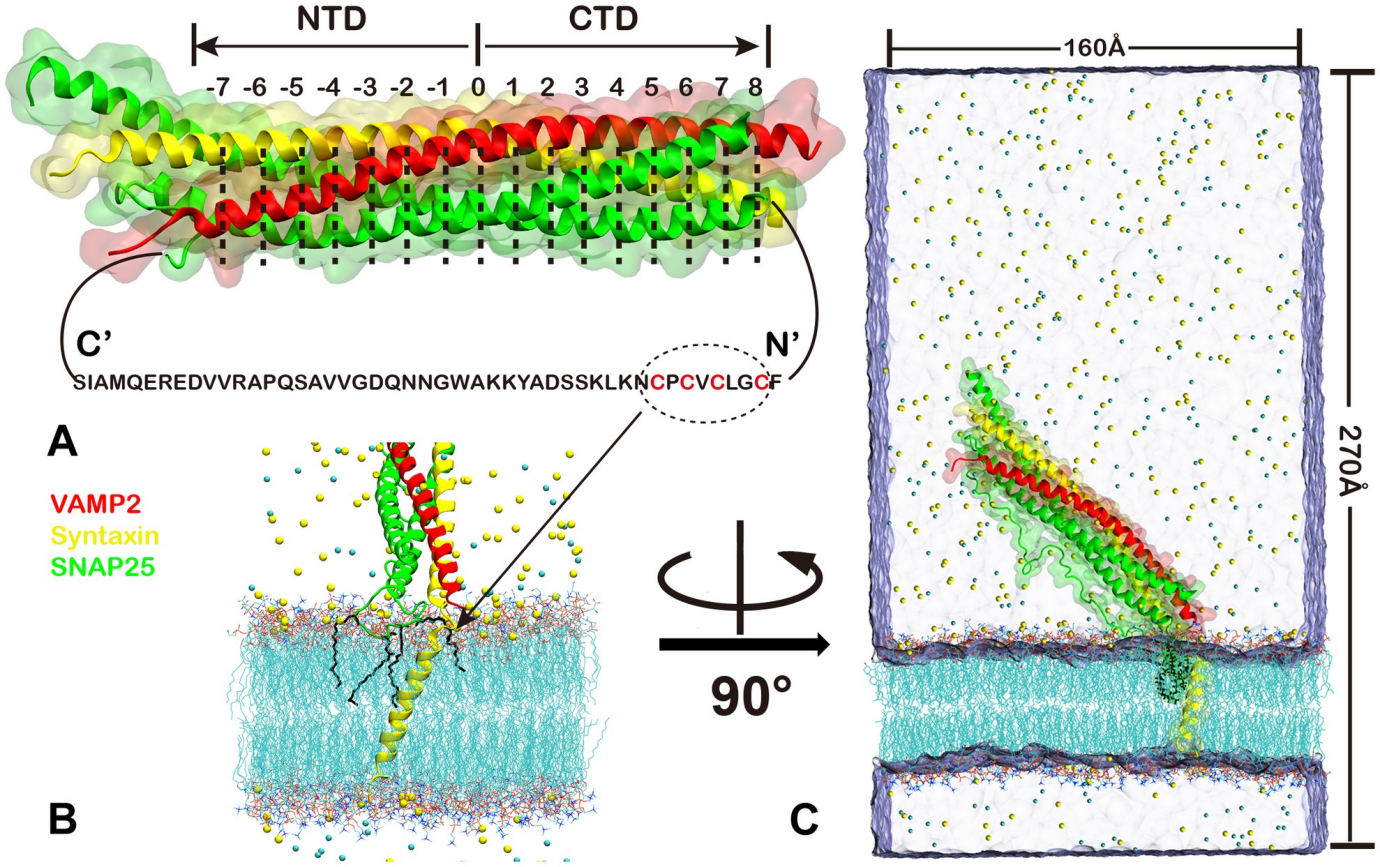
SNARE complex and the simulation setup. (A)Schematic of ternary SNARE four helix bundle with Syntaxin(yellow), VAMP2(red), SNAP25(green). The notation of SNARE layers is indicated. The sequence of linker loop of SNAP25 is shown, 4 palmitoylated cysteines anchored to the membrane were shown in red. (B)Assembly of SNARE complex with the membrane, with the palmitoylated cysteines on SNAP25 highlighted. (C)The initial simulaiton system. The SNARE core domain is placed ~ 40° reletive to the membrane.

The material exchanges across cell membranes or membrane-bounded organelles such as neural transmitter release and hormone secretion are mediated by the soluble N-ethylmaleimide-sensitive factor attachment protein receptor (SNARE) complex[1–3]. This minimal neuronal exocytotic fusion machinery composes a four-helix bundle formed by trans-membrane protein vesicle-associated membrane protein 2 (VAMP2, also called Synaptobrevin), plasma membrane protein Syntaxin and SNAP-25, VAMP2 and Syntaxin each provides one helix to the SNARE four helix bundle, and the other two helixes are contributed by SNAP-25 (Fig 1A). VAMP2 and Syntaxin are anchored to the vesicle membrane and plasma membrane respectively via their C-terminal transmembrane portion, and SNAP-25 is tethered to plasma membrane by four palmitoylated cysteines located at its linker loop (LL) region[4–6] which connects its N- and C- terminal SNARE motifs. The zipping process of VAMP2 with Syntaxin and SNAP25 provides the pulling force to overcome the energy barrier for the two membrane come into proximity[7] and initiates membrane fusion. Membrane fusion is a precisely controlled process, the zippering of the SNARE protein is modulated by auxiliary proteins such as Munc-18 and Munc-13[8]. The fast fusion process also couples with action potential regulation by Ca^2+^ sensor Synaptotagmin[9] and by Ca^2+^ sensors associated complexin[10]. All these factors work cooperatively and lead to heterogeneity of efficiency in different cells.

In prior to the SNARE complex formation, binary complex of Syntaxin and SNAP25 may be formed on plasma membrane[11–13], but their exact conformations and clustering states, which are affected by the presence of membrane[14], are still open questions and remain unclear. It is suggested that the Syntaxin-SNAP25 complex need to be converted into Munc18 stabilized close Syntaxin which is opened by Munc13 to form Munc18 and Munc13 bounded trans-SNARE complex. The Munc18 and Munc13 bounded trans-SNARE complex correspond to the final assembly with VAMP2 for SNARE complex[8]. In neural cells, the VAMP2 is primed to the pre-structured binary trans-SNARE complex by the Ca^2+^ stimulus signal and results in fast response of neurotransmitter release. When the stimulated initial signals are transmitted, the pre-structured trans-SNARE complex might pose standing or lying down conformations which are promoted by Synaptotagmin and complexin. The nucleation of SNARE complex started when VAMP2 contacts on the N-terminal side of trans-SNARE complex then processed to the C-terminal side[15], forces the approaching of synaptic vesicles to the cell membrane, at last leads to the hemi-fusion stalk formation and the fusion pore formation. The membrane-proximal linkers between the transmembrane region and SNARE helix of Syntaxin and VAMP2 contact finally in the membranes for additional energy providing [7].

The pre-fusion state of Syntaxin bound to the membrane including a segment of SNARE motif, membrane-proximal linker and transmembrane^™^ regions had been determined by NMR[14], the solution structures reveal different conformations of Syntaxin other than these observed in SNARE complex and in the absence of cell membrane. In the NMR structures, the membrane-proximal linker region indicates a disordered nature, while the post-fusion state of Syntaxin determined with VAMP2 in SNARE complex shows continuous helix on this region and implies standing conformation. How zippering of SNARE complex provides energy for membrane fusion is not fully understood, a half-zippered SNARE complex structure is suggested to represent a functional intermediate in the fusion process [16]. Moreover, the linker loop (LL) between the two SNAP25 SNARE domains was proved to have the ability to improve the speed of SNARE assembly, although the region has not been determined in any structural studies due to its high flexibility[17–19]. The four Cysteine residues located near the CTD region are palmitoylated and serve as anchors to tether the protein onto the membrane.

The zipped core region of SNARE is a coiled-coiled four parallel helices bundle that composes of 15 conserved hydrophobically stacked layers within which an ionic layer 0 divides the complex into C-terminal domain (CTD) and N-terminal domain (NTD) (Fig 1A)[4]. The zipping/unzipping dynamics and its force regulation have been studied by single molecule experiments using optical tweezers[20] and magnetic tweezers [21] and several intermediate states have been identified [20, 22]. Also steered molecular dynamics (SMD) simulations have also been preformed to probe the details of the unzipping process and suggest the possible unzipping pathway at the atomistic level [23, 24].

Although successes in understanding the mechanisms of SNARE formation and force generation have been achieved, there remain questions unanswered. Especially, SNARE formed between the junction of cell membrane and synaptic vesicles, the dynamics and kinetics of SNARE complex are inevitably influenced by the presence of membrane. To address how membrane may affect the dynamics of SNARE complex and its unzipping pathway, we performed molecular dynamics (MD) simulations of SNARE complex in the membrane environment and with the LL of SNAP25 tethered to the membrane via palmitoylated cysteines. Several successful studies using all-atom simulation[19, 25] or coarse grained method[26–28] on SNARE system had been conducted and provided much valuable results in various aspects, such as the SNARE-mediated membrane fusion[27] and interactions of SNARE with complexin[29].

## Methods

### System setup

We started our simulations using crystal structure (PDB:1SFC)[4] as the initial SNARE core conformation, and the NMR structure of Syntaxin trans-helix [14] as transmembrane and linker conformation. The initial configuration of the missing LL loop of SNAP25 was generated by Amber Tools. We combined all these segments manually with VMD[30] and refined the final structure with NAMD[31]. CHARMM36 force field[32] was used to describe the atom interactions of the system.

Two pre-equilibrated membrane models were used in our simulations. In both models, the ratio of CHOL to the glycerol lipids are 1:3, which is close to the ratio in most real cell membrane. One with POPE POPS POPC by the proportion of 2:1:1:1 (MEM1), which is close to the ratio in the inner leaflet of the cell membrane. Another with all glycerol lipids are PIP2 (MEM2), because it is suggested that high accumulation of PIP2 was required for membrane fusion[33]. The four cysteines in SNAP25 LL region were modified to link palmitic acid covalently and inserted into the membrane (Fig 1B). The unavailable parameters for palmitoylated cysteines were used from picking these for similar functional groups in CGenFF[34]. The TIP3P water model[35] were used to solvated the system and and Na^+^, Cl^−^counterions were added to neutralize the system with additional 0.15M salt concentration to mimic the physiological conditions. Due to the flexibility of membrane proximal linker domain of Syntaxin in pre-structure state, we have modeled the whole complex with two different conformations on this the membrane-proximal region of Syntaxin, i.e. residues 258 to 260. In one model, this region adopts loose helical conformation and extends the transmembrane helix, which may induce higher stress when connecting to Syntaxin SNARE motif on its N-terminal, thus we named it as high stress (HS) model. In another model, we use an unstructured loop for residues 258 to 260 to connect the N- and C-terminals, and we named this model as low stress (LS) model. Initially, the SNARE core domains were placed onto the membrane with an angle about 40° (Fig 1C). A detailed list of systems and simulations are shown in Table 1.

**Table 1:** Summary of MD simulations performed.

Name	Description	Velocity (nm/ns)	Atoms	Time(ns)
**Free1**	HS-MEM1	-	229,357	100
**Free2**	LS-MEM1	-	192,725	100
**Free3**	HS-MEM2	-	237,360	100
**Free4**	Without MEM	-	85992	96
**Free5**	Without MEM	-	85992	78
**FU1**	HS-MEM1	0.5	338,325	85
**FU2**	LS-MEM1	0.5	331,470	85
**FU3**	HS-MEM1	0.5	338,325	85
**FU4**	LS-MEM1	0.5	331,470	85

Three independent free MD simulations are represented as Free1, Free2 and Free3. HS stands for high stress model, and LS stands for low stress model, which are the two initial conformation of our simulation. MEM1 indicates the use of lipid membrane model with POPE POPS POPC CHOL by the proportion of 2:1:1:1 and MEM2 the use of lipid membrane model with PIP2 CHOL by the proportion of 3:1. As a comparison, two free MD simulations without membrane (and without CYS palmitoylation) were also performed. Force induced unzipping simulations by SMD were represented by FU, four simulaitons (FU1, FU2, FU3 and FU4) are carried out.

### Energy minimization and equilibration

We minimized and equilibrated the three systems with standard simulation protocols. At first, each system was energy minimized carefully in a multiple-step process, i.e. keeping protein and lipids constrained first, then keeping protein constrained, keeping protein backbone constrained and finally all free. The resulted systems were then subjected with NPT equilibration for 1ns with protein constrained and then 5ns without any constraint. In these steps, the waters of each system were kept out of the membrane using a script provided in NAMD membrane protein tutorial and the integration time step of 1 fs. Periodic boundary condition was used in all the simulations.

After initial energy minimization and equilibration steps, additional 100ns production runs were performed for all systems with 2 fs integration time step and keep the water rigid. In all simulations we use PME method[36, 37] for the calculation of long range electrostatic and 12Å cut-off for short range nonbonded interactions. Langevin dynamics was used for temperature control at 310K and pressure was maintained at 1atm using Nose-Hoover-Langevin piston method[38].

### Steered molecular dynamics (SMD) simulations

To investigate the unzipping pathway of SNARE complex in presence of membrane, we performed SMD simulations using the above equilibrated systems as starting configuration. Because we do not observe any major significance in the simulation with PIP2 consisting membrane, The SMD simulations are only performed with the systems of MEM1. For each such system, snapshot at 100ns is chosen. Thus, two independent initial configurations are used. Additional waters are added to each configuration to enlarge the system to accommodate unzipping of the SNARE complex, and additional counterions are also added (Fig 1C). The results system was then subjected to 10 ns of equilibration under 1 fs time step. The 5ns and 10ns snapshots for each system were used in SMD simulations, results in four forced unzipping trajectories. With 8 phosphorus atoms on lipids at the boundary of the membrane constrained at the initial positions, the C terminus of VAMP2 is pulled with a dummy spring of spring constant ~ 70 pN/nm, the pulling velocity is 0.5 nm/ns. All analyses and graphics shown in this paper were performed with VMD[30].

## Results

### Effects of membrane on the dynamics of SNARE complex

SNARE complex formed at the junction of cell membrane and synaptic vesicles, the formation process may also be affected by the presence of membrane. To check how the dynamics of SNARE is influenced by the membrane, we performed large-scale MD simulations with the lipid membrane included. In the simulations, we also included the link loop (LL) region of SNAP25 which is missed in all related structures (Fig 1A), the LL region is tethered to the membrane via four palmitoylated cysteines located closely to the N-terminal SNARE motif of SNAP25 (Fig 1B). At first, the transmembrane helix of Syntaxin was inserted into a pre-equilibrated membrane system with lipid composition mimicking the intact cell membrane. Then the SNARE core domain was placed with an angle ~ 40° relative to the membrane (Fig 1C), the ~40°.orientation is chosen to deviate away from parallel or perpendicular setup, which will help us check in the simulations whether SNARE/membrane interactions can control SNARE orientation. At last, the linker region between transmembrane helix and the SNARE motif was added using the NMR structure as template[14]. Considering the relative high stress of the Syntaxin linker region in the modeled structure, which may hinder the formation of contacts between membrane and SNARE, we build two different models, with relaxing the first exposed helical circle manually on the transmembrane helix of Syntaxin (Free2) or not (Free1). Moreover, to check the effects of membrane lipid composition, we uses two different membrane models in our simulation. In the first model(MEM1), the membrane components are mimicking the cell membrane at the resting state. Because it has been suggested that PIP2 accumulates at the membrane fusion site[33], we also use a PIP2 membrane which only consists PIP2 and cholesterol (Free3). The simulations we performed were summarized in Table 1.

**Fig 2:**
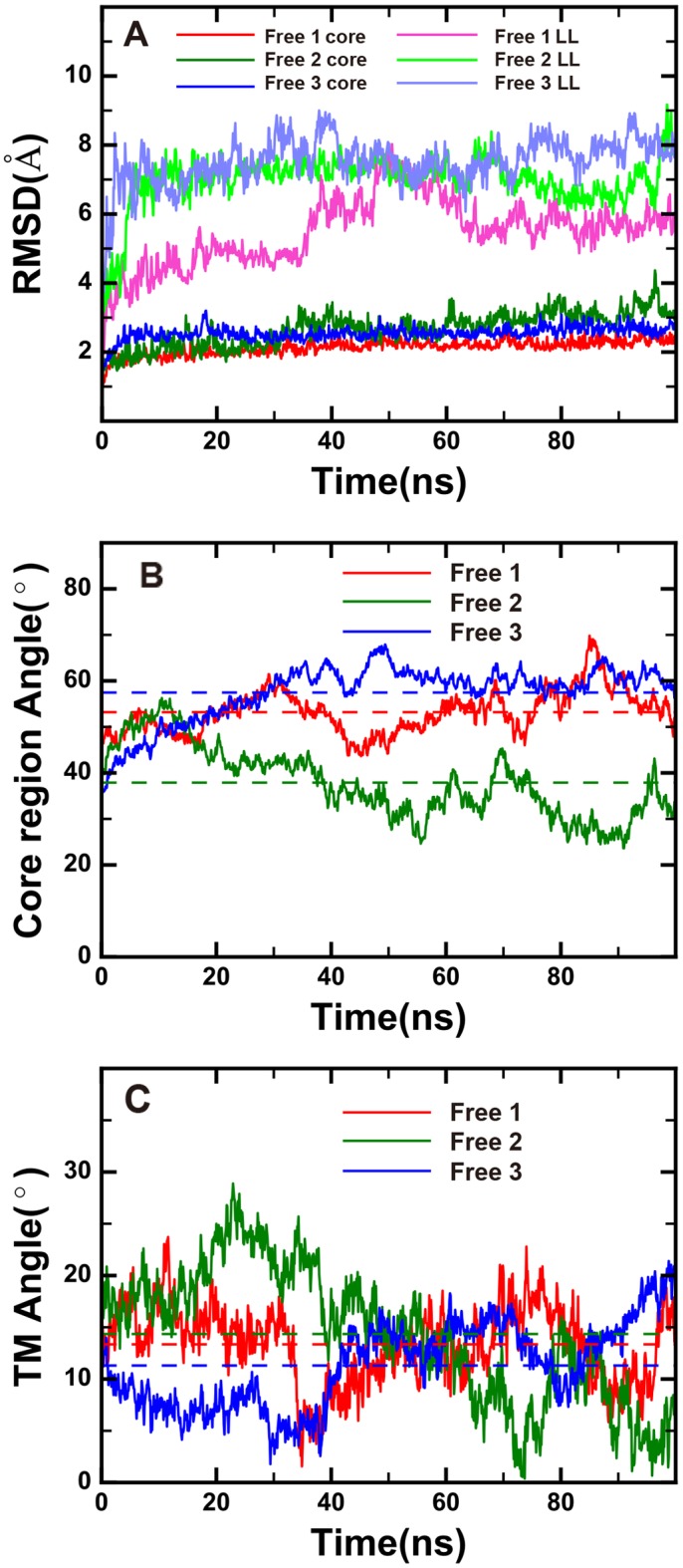
The free molecular dynamics simulations. (A)The RMSD of three different simulations, resutls for SNARE core domain and LL region are shown. (B)The change of the angle between SNARE core domain and the membrane. (C)Change of the angle between the transmembrane domain of Syntaxin and the membrane.

The time-courses of root mean square deviation (RMSD) in our 100ns free dynamics simulations for the core domain and the SNAP25 LL are shown in Fig 2A, which indicate that the SNARE core domain is rigid (RMSD lower than 3Å most of the times), these results are also in good agreement with previous MD studies without membranes[19, 25]. On the contrast, the SNAP25 LLs differ a lot from the modeled structures (RMSD greater than 5Å).

The changes of the angle between core SNARE complex and the membrane for our simulations are shown in Fig 2B. This angle is controlled by the membrane-proximal region of Syntaxin and the membrane tethered region of LL in SNAP25. The angle decreases to around 30° in simulation Free2, but increased to 50~60° in simulations Free1 and Free3. During the simulations, interactions between SNARE core region and membrane mainly focused on its CTD bottom besides the anchoring points. The CTD side of SNARE core domain consists several positively charged residues which can form charge-charge interactions with the negatively charged PS or PIP2 lipids in the membrane. But the interaction between the NTD side of SNARE core domain and membrane is not large enough to change the SNARE orientation to make it parallel (if the interaction is attractive) or perpendicular (if the interaction is repulsive) to the membrane. It is believed that the SNARE core should locate parallel and very close to the membrane during some steps in the process of fusion pore formation[26, 33, 39] and perpendicular to the membrane after the vesicle fusion[7]. These conformations may result from other factors, not due to properties of the membrane.

In the initial models, the transmembrane helix of Syntaxin is placed with ~10° relative to the vertical of the membrane, and we did not observe any significant changes in all our simulations (Fig 2C), which is different with the case for VAMP2, which might have a high tilting angle[40].

**Fig 3:**
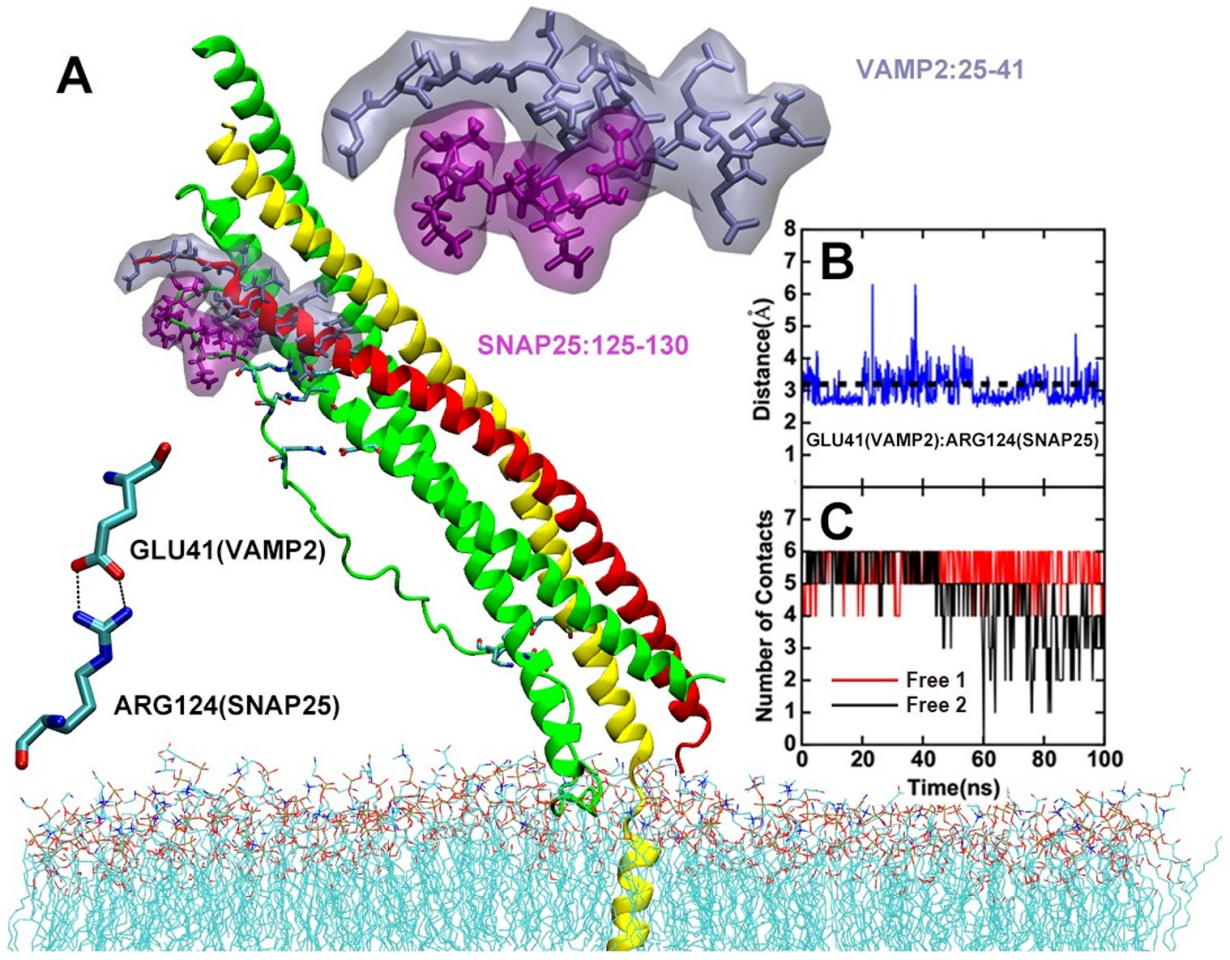
LL region of SNAP25 forms interactions with VAMP2. (A)snapshot of the free MD simulation. The interactions on the N-terminal residues of NTD region are highlighted, with VAMP2 residues 25 to 41 shown in gray and SNAP25 residues 125–130 shown in purple. A specific salt bridge between VAMP2 E41 and SNAP25 R124 is also indicated. (B)The time-course of the distance VAMP2 E41 and SNAP25 R124 for Free2, a distance below 3.2Å can be viewed as salt bridge fromed. (C)Number of contacts between of SNAP25 with VAMP2 residues 24 to 41.

In our simulations, LL of SNAP25 is observed to show fast and transit interactions with SNARE core complex, especially at the SNARE NTD region (CTD of the LL region). Several pairs of salt bridges formed and broke in quick dynamical equilibrium (Fig 3A). Among which, the relative stable salt bridge between residue E41 of VAMP2 and R124 of SNAP25 LL (Fig 3A and 3B) might be important. Since SNARE complex formation starts from the NTD region by the initial interaction between t-snare and VAMP2 then zipped toward the CTD. The E41/R124 recognition may help t-snare to recognize and stabilize VAMP2 for later zipping process. Other contacts between LL and VAMP2 NTD motif are also observed and the change of number of contacts along the simulations is shown in Fig 3C. These contacts may cooperatively assist SNARE NTD nucleation process. To role out the possibility that these observed interactions are resulted by the presence of membrane, we also performed simulations with the LL in the absence of membrane (Free4 and Free5). In both simulations, similar interactions are also observed, indicating that these interactions are indeed resulted by the presence of LL (S1 Fig).

### Dynamics of SNARE core region

The SNARE core region composed 15 hydrophobic layers (layer -7 to -1 and 1 to 8) and 1 ionic layer (layer 0) (Fig 1A), the zipping process of these layers provide energies to drive membrane fusion, single molecule studies indicate that the zipping process of the SNARE formation releases ~68 *k*_B_T of free energy (~35 *k*_B_T for NTD zipping, ~28 *k*_B_T for CTD zipping and additional energy from the linker region) [20]. The dynamics of SNARE core complex alone has been studied previously by MD simulations [19, 25]. In our current study, membrane and LL of SNAP25 are included, which result in differences on SNARE dynamics with previous studies, especially in the SNARE CTD region.

**Fig 4:**
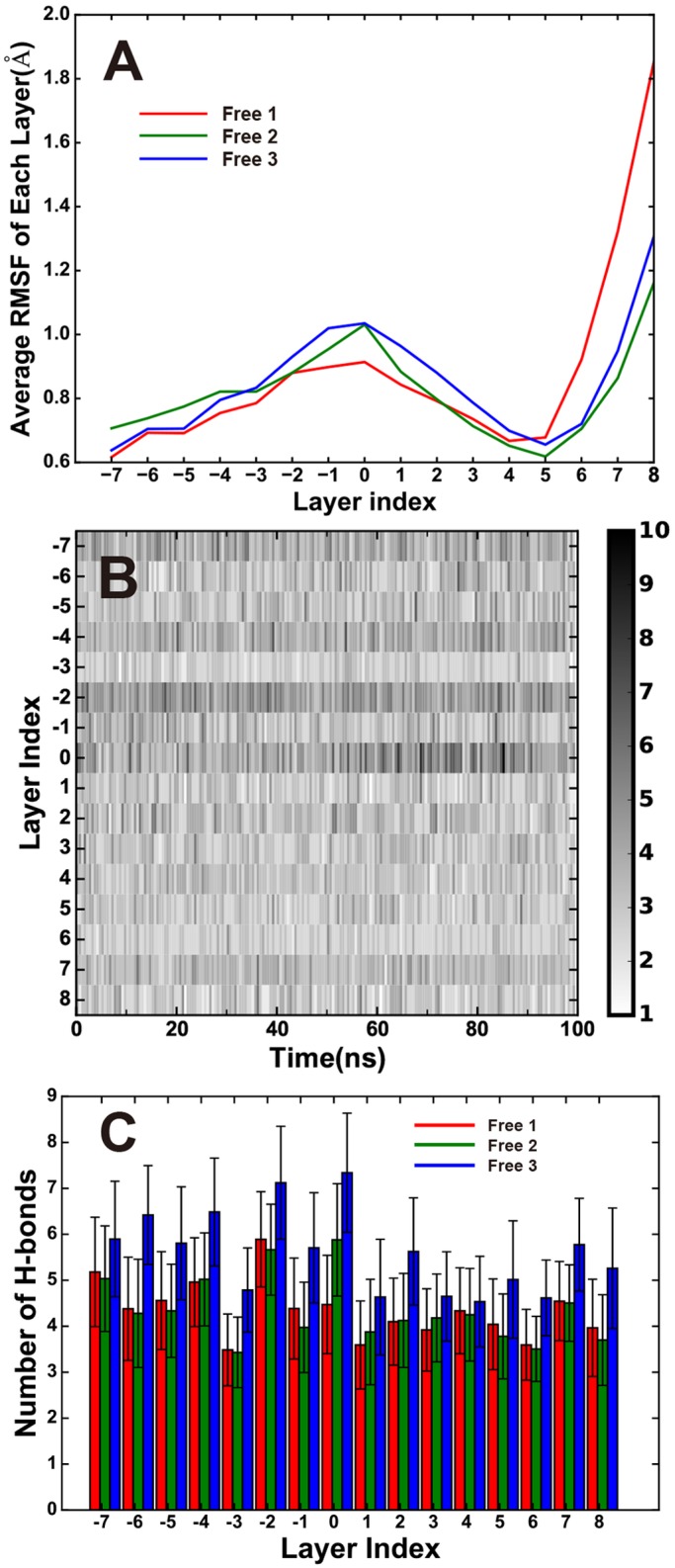
The dynamics of each SNARE layer. (A)Averaged root mean squared fluctuation of each layer in the simulations. (B) Time-course of the numbers of hydrogen bond between the layer residues with their surroundings for each layers. (C)Average number of Hydrogen bond between the layer residues with their surroundings for the last 20 ns of the two simulation (80–100 ns).

The averaged root mean square fluctuations (RMSF) of each SNARE layer for all three simulations are shown in Fig 4A, the results for all simulations are similar. Interestingly, the ionic layer (layer 0) and the C-terminal layers (layer 7 and 8) display larger fluctuation, especially the C-terminal-most layer (layer 8), indicating the relative flexibility of CTD than that of NTD and in agreement with the experimental results that zipping of NTD release more free energy.

We also calculated the time-courses of the number of H-bonds with the surroundings (Fig 4B) and the average number of H-bonds formed in 80-100ns for each layer (Fig 4C), the NTD layers (-7 to 0) show slightly higher numbers of hydrogen bonds in all simulations, again suggesting it is more stable than the CTD layers. The zero layer contains most conserved residues among SNAREs, the map for the number of H-bonds (Fig 4B) shows a clear boundary on this layer between NTD and CTD. This is also in good agreement with the single-molecule force spectroscopy experiments which reveal the existence of intermediates between NTD and CTD.

### Unzipping SNARE with force in the presence of membrane

Previous Steered MD (SMD) studies with implicit water model show the unzipping of SNARE complex undergoes different pathways, either or both VAMP2 and Syntaxin can be disrupted, which is not consistent with the single-molecule force spectroscopy measurement. It is argued that the differences come from the several orders of magnitude differences in time scales between the simulation and the experiments. Similar results are also obtained in our explicit water MD studies using CHARMM 27 force field and soft spring constant compared with previous studies without membrane and SNAP LL region (S2 Fig). In real vesicle fusion process, the SNARE zipping pathway is restricted by the membranes, thus the differences may come from the absence of membrane in previous simulations. To test how SNARE formation maybe regulated by membrane, we performed SMD studies for unzipping the SNARE complex to check the influences on the reaction pathway by membrane.

**Fig 5:**
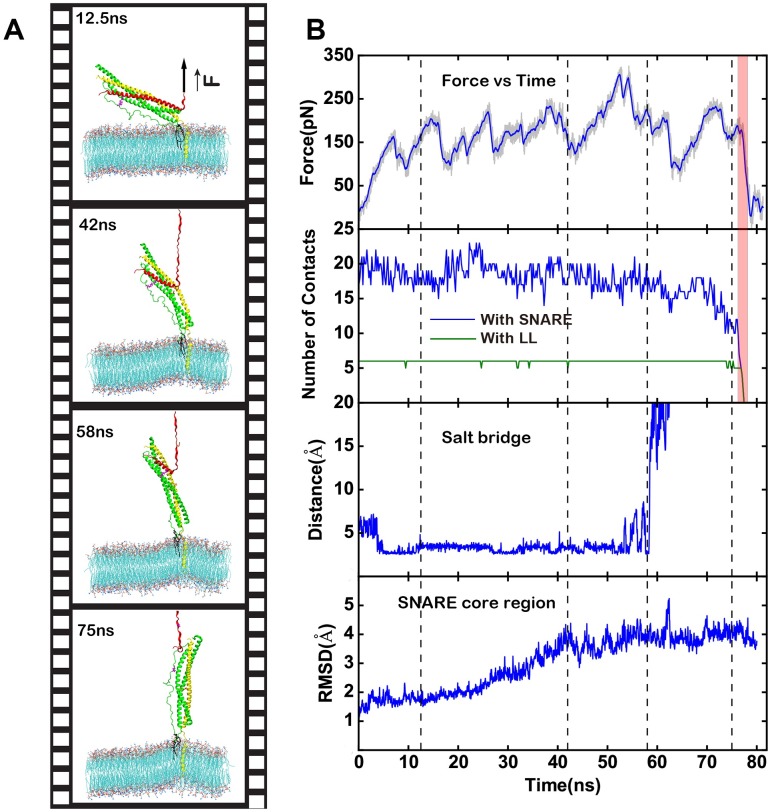
Steered molecular dynamics simulations. (A)Snapshots for a representative unzipping simulation (FU2). (B)First panel: the force curve. Second panel: the number of contacts between VAMP2 residues 25 to 41 with SNAP 25. Third panel: the distance between VAMP2 E41 and SNAP25 R124 salt bridge. Fourth panel: the RMSD of the rest part of SNARE core domain when VAMP2 is pulled away. The pink area indicated the final detachment between VAMP2 and the rest of SNARE core domain, four dashed line represents the time of snapshots in (Fig 5A).

In our SMD simulations, 8 phosphorus atoms on lipids at the boundary of the membrane were constrained and the C-terminal of VAMP2 is pulled with a dummy spring (spring constant ~ 70pN/nm) moving at constant velocity ~ 0.5 nm/ns. A total of 4 SMD simulations starting from different initial configuration (FU1 to FU4) were carried out (Table 1). The highest force peaks for the simulations range from 300pN to 400pN. Snapshots for a representative SMD trajectory (FU1) are shown in Fig 5A. In all our simulations, unlike previous simulations without membrane [23] VAMP2 dissociates from the rest parts of SNARE sequentially from CTD to NTD. The disruption of one layer or multiple layers at the same time gives rise to a force peak (Fig 5B, first panel). The number of contacts between VAMP2 and the rest SNARE decreases following the disruption of each layers (blue in Fig 5B, second panel), while that between VAMP2 and LL of SNAP25 was not affected until the final dissociation of the system (green in Fig 5B, second panel). The observed E41/R124 salt bridge breaks at ~58 ns after the -4 layer of SNARE complex disrupted (Fig 5B, third panel). The resting part of SNARE also changes upon the VAMP2 unzipping away, its RMSD remains unchanged (less than 2Å) before the disruption of layer +8, but increases gradually to about 4Å when layer 0 is disrupted, and remains unchanged thereafter (Fig 5B, fourth panel). The pink shaded area in Fig 5B indicates the final detachment of the VAMP2 with the rest part of SNARE domains.

**Fig 6:**
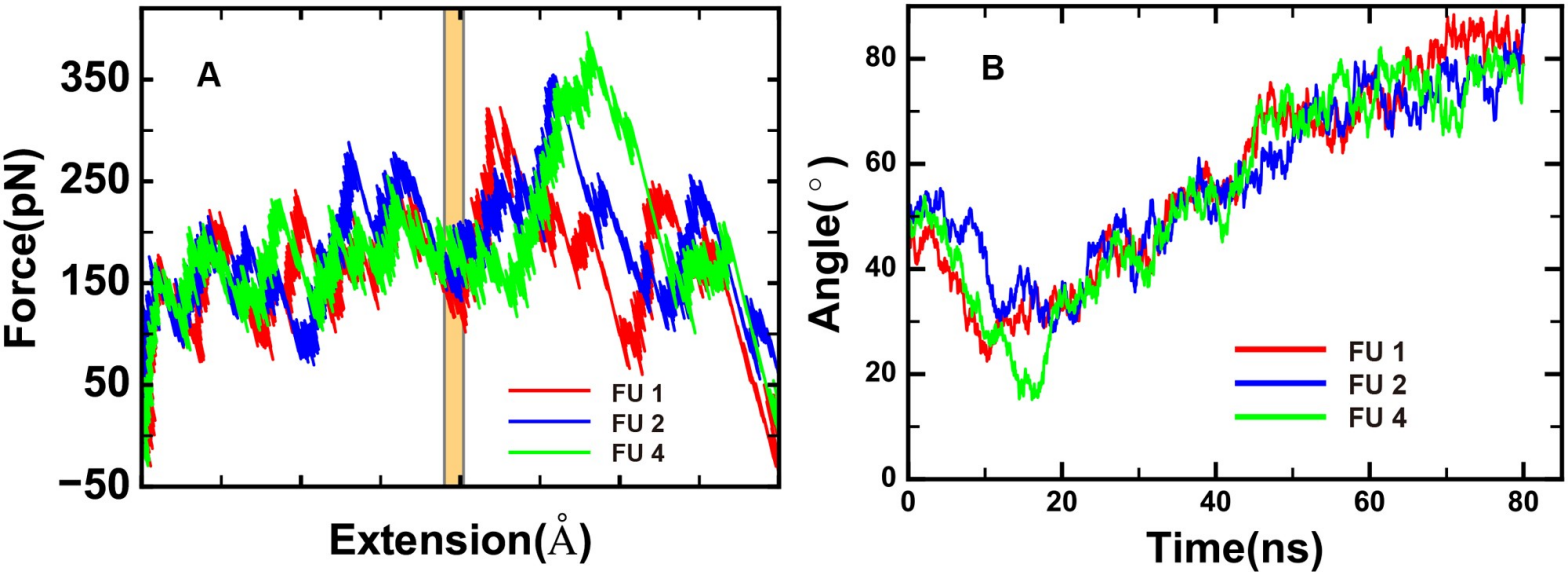
Analysis of SMD simulations. (A)Force-extension curves of SMD simulations FU1, FU2 and FU4. The yellow, shaded area represents zero layer position. (B)The change of the SNARE orientation with the membrane during the simulations.

The force-extension curves of three simulations (FU1, 2 and 4) are shown in Fig 6A. The three simulations show similar unzipping pathway in a layer by layer manner prior to the disruption of zero layer (indicated by the yellow shaded area), but differ a lot after that where multiple layers can be disrupted at the same time which results the highest force peak. The force required to disrupt a layer is higher in the NTD side than that in the CTD side, consists with the free dynamics simulations where NTD is more stable. The pathway for simulation FU3 is different, in this simulation, a twist on SNAP25 is observed, and the unzipping pathway is significantly altered (S3 Fig).

When VAMP2 was unzipped away, the resting part of SNARE core domain changes its orientation. The angle between the resting part of SNARE core domain and the membrane is shown in Fig 6B. After the force is applied, the angle decreases first (before 10–20 ns and the disruption of layer 6) and the SNARE complex is more close to parallel to the membrane. At the time, SNARE tilts because the pulling force mainly affects the bottom of CTD and the C-terminal layers +8 and +7 act as pivots which bend the SNARE. This result suggests that the zipping force at CTD may facilitate the orientation change of SNARE complex to enable the approaching of vesicle toward the cell membrane.

These results in certain degrees are in consistent with the single molecule force spectroscopy experiments, which revealed the existence of several intermediate states which approximately located in the zero layer [21, 22, 27].

## Discussion

Membrane plays important roles in the process of vesicle fusion. In our studies, we included the membrane in MD simulations which are omitted previously [19, 23–25]. We also included the LL region of SNAP25 which not only provide additional anchoring for SNARE machine to tether onto the membrane, but may also affect the integrity of the whole SNARE complex.

Our simulation results indeed indicated that the NTD of SNARE core domain is more stable than the CTD side. Moreover, the simulations suggests that the attraction (or repulsion) between SNARE core region and the cell membrane is much weak than the strong coiled-coil interactions of SNARE motifs, regardless the lipid composition of the membrane. Thus, the membrane may only provide spatial constrains on SNARE formation. In the presence of membrane, VAMP-2 always unzips sequentially from the Syntaxin/SNAP25 core, unlike two different unzipping processes (VAMP-2 or Syntaxin) observed in previous simulations, suggesting that the membrane also stabilize the interaction between Syntaxin and SNAP25. We should note here that several important factors important to the fusion process are not considered in our studies, such as MUNC proteins, complexin and Synaptotagmin, these molecules may bridge the interaction between the SNARE complex and the membrane, as confirmed by recent studies[39, 40], these factors together account for the founding that curvature stress of membrane can affects SNARE-mediated liposome fusion[41, 42].

Our simulations suggest that the LL region of SNAP25 interacts directly with VAMP2, thus may provide additional mechanical strength for the initial SNARE nucleation from the NTD side. This region may also play positive roles during the zipping process, it tethers the t-SNARE complex firmly to the membrane through palmitoylated cysteines and prevents the dissociation of SNAP25 and Syntaxin under force (as observed in previous MD simulations studies where separation of Syntaxin with the rest part of SNARE domain is observed). These results provide an alternative explanation to previous studies where the different redox states of these cysteines are found to be important [19].

In conclusion, our MD results provide atomistic explanation to understand the single-molecule force spectroscopy experiments. The SNARE unzipping under force can be considered as the reverse process of SNARE formation. The zipping of SNARE from NTD to CTD releases large amount of energies and generates the force to pull the membrane and vesicle together. Also, the zipping process may correlate with the orientation change of SNARE complex to release special constraints for membrane fusion. The interactions between the SNAP LL and the SNARE core domain may help stabilizing initial SNARE nucleation process and assist SNARE formation. Single molecule force spectroscopy experiments involving the LL region in the presence of the membrane should be able to confirm the role of membrane and the importance of LL/SNARE core interactions.

## Supporting information

S1 Fig Free simulations with LL in the absence of membrane. (A) Averaged root mean squared fluctuation of each layer; (B) The time-course of the distance VAMP2 E41 and SNAP25 R124 for Free4; (C) Number of contacts between of SNAP25 with VAMP2 residues 24 to 41 in Free4 and Free5.
(TIF)

S2 Fig Simulations of the force unzipping without membrane and LL. The initial structure used for unzipping simulation without membrane and LL are shown in the left panel, while the right panel is a snapshot of the forced unzipping simulation.
(TIF)

S3 Fig Simulation trajectory for FU3. (A) Force-extension curve. (B) The tilting of SNAP25. The C-terminal motif of SNAP25 loses its interaction with Syntaxin and follows the movement of VAMP2. The intact conformation of SNARE CTD is shown transparently for comparison.
(TIF)
