# Supplementary figures and images for: The influence of cell membrane and SNAP25 linker loop on the dynamics and unzipping of SNARE complex
Source: PLoS One. 2017 Apr 20;12(4):e0176235. doi: 10.1371/journal.pone.0176235 (PMC5398687; doi:10.1371/journal.pone.0176235)

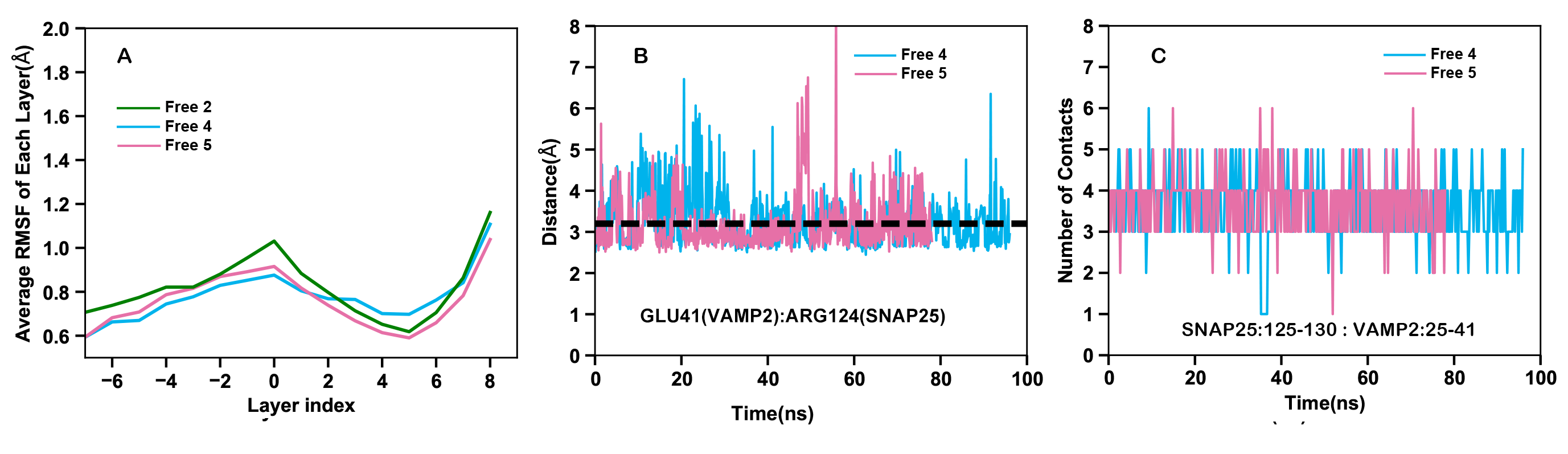

Supplement: S1 Fig — (A) Averaged root mean squared fluctuation of each layer; (B) The time-course of the distance VAMP2 E41 and SNAP25 R124 for Free4; (C) Number of contacts between of SNAP25 with VAMP2 residues 24 to 41 in Free4 and Free5. (TIF) [file pone.0176235.s001.tif]

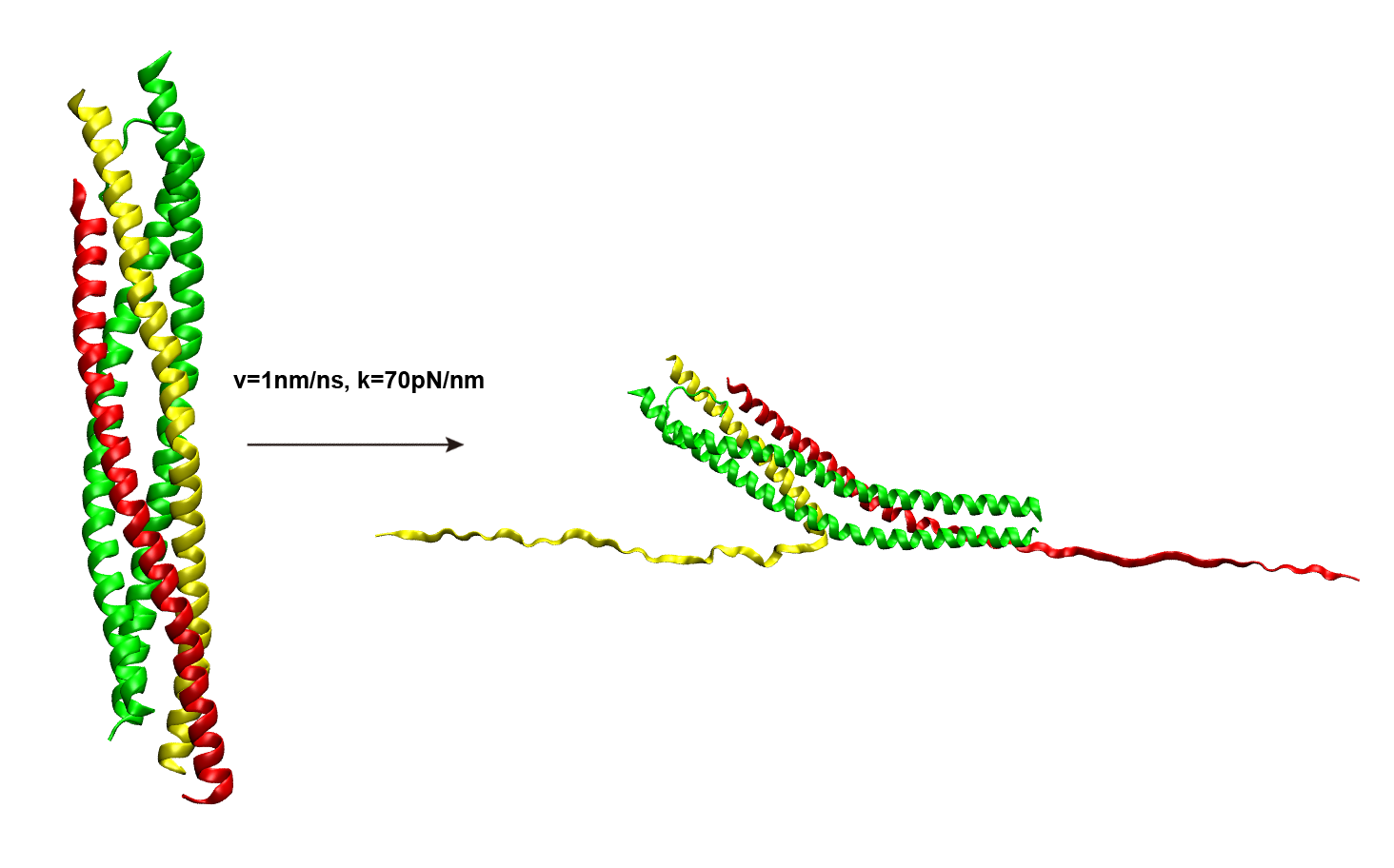

Supplement: S2 Fig — The initial structure used for unzipping simulation without membrane and LL are shown in the left panel, while the right panel is a snapshot of the forced unzipping simulation. (TIF) [file pone.0176235.s002.tif]

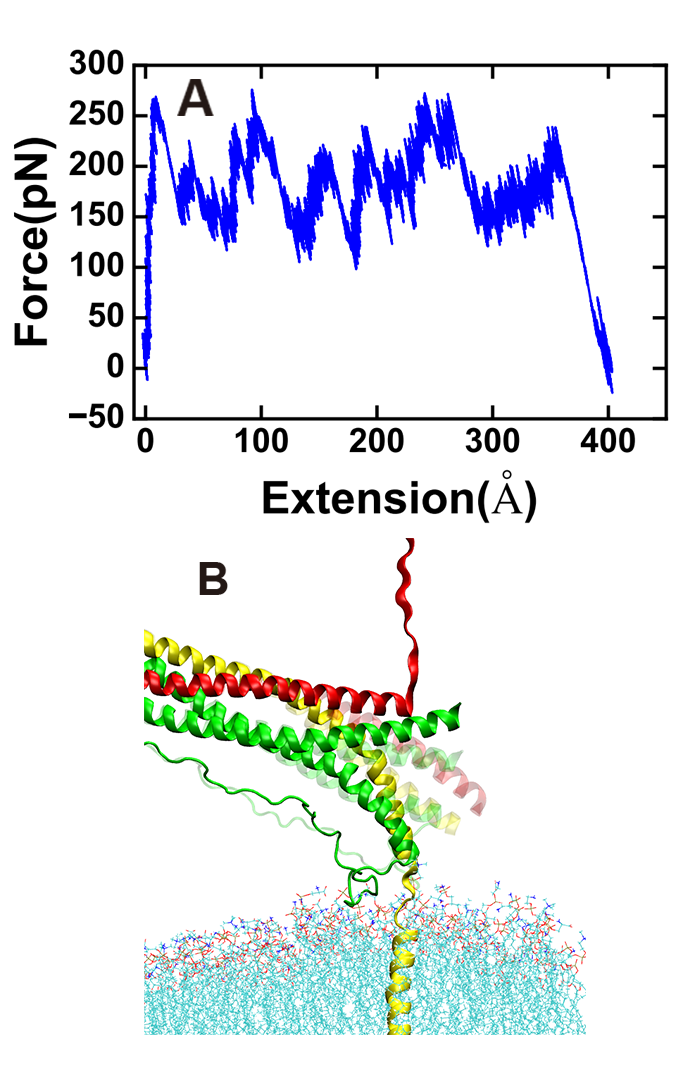

Supplement: S3 Fig — (A) Force-extension curve. (B) The tilting of SNAP25. The C-terminal motif of SNAP25 loses its interaction with Syntaxin and follows the movement of VAMP2. The intact conformation of SNARE CTD is shown transparently for comparison. (TIF) [file pone.0176235.s003.tif]
